# Training interval in cardiopulmonary resuscitation

**DOI:** 10.1371/journal.pone.0226786

**Published:** 2020-01-16

**Authors:** Marilyn H. Oermann, Michael A. Krusmark, Suzan Kardong-Edgren, Tiffany S. Jastrzembski, Kevin A. Gluck

**Affiliations:** 1 School of Nursing, Duke University, Durham, North Carolina, United States of America; 2 L3 Technologies at the Air Force Research Laboratory, Wright-Patterson Air Force Base, Wright-Patterson AFB, Ohio, United States of America; 3 Center for Medical Simulation, Boston, Massachusetts, United States of America; 4 711^th^ Human Performance Wing, Airman Systems Directorate, Air Force Research Laboratory, Wright-Patterson Air Force Base, Wright-Patterson AFB, Ohio, United States of America; University of Palermo, ITALY

## Abstract

**Aim:**

Although evidence supports brief, frequent CPR training, optimal training intervals have not been established. The purpose of this study was to compare nursing students’ CPR skills (compressions and ventilations) with 4 different spaced training intervals: daily, weekly, monthly, and quarterly, each for 4 times in a row.

**Methods:**

Participants were nursing students (n = 475) in the first year of their prelicensure program in 10 schools of nursing across the United States. They were randomly assigned into the 4 training intervals in each of the schools. Students were trained in CPR on a Laerdal Resusci Anne adult manikin on the Resuscitation Quality Improvement (RQI) mobile simulation station. The outcome measures were quality of compressions and ventilations as measured by the RQI program.

**Results:**

Although students were all certified in Basic Life Support prior to the study, they were not able to adequately perform compressions and ventilations at pretest. Overall compression scores improved from sessions 1 to 4 in all training intervals (all p < .001), but shorter intervals (daily training) resulted in larger increases in compression scores by session 4. There were similar findings for ventilation skills, but at session 4, both daily and weekly intervals led to better skill performance.

**Conclusion:**

For students and other novices learning to perform CPR, the opportunity to train on consecutive days or weeks may be beneficial: if learners are aware of specific errors in performance, it may be easier for them to correct performance and refine skills when there is less time in between practice sessions.

## Introduction

The association between high quality cardiopulmonary resuscitation (CPR) and patient survival has been well documented over the years. High quality CPR that meets the guidelines of the American Heart Association (AHA) and European Resuscitation Council improves patient outcomes and chances of survival following a cardiac arrest [1–7]. Many health care providers, however, are unable to perform high quality CPR even though they satisfied CPR training requirements [1, 8–11].

Research has repeatedly documented a significant decay in CPR skills within weeks to months after training [1, 11–16]. Thus, CPR training that is completed in a 1- or 2-day course may confirm providers’ acquisition of knowledge and skills at that moment, but those skills deteriorate rapidly without continued practice. Based on a review of studies on resuscitation education, investigators concluded that increasing the frequency of training would improve training efficacy, prevent skill deterioration, and improve CPR skill performance [1].

Spaced or distributed practice involves frequent, short training sessions over a period of time. With spaced training, learners have guided practice spread across several training sessions. Studies support that spaced CPR training improves performance and retention of skills [1, 12, 15, 17–19].

In addition to the burgeoning evidence regarding the advantages of distributed practice for knowledge and skill retention, there is an extensive literature in the cognitive sciences about the benefits of feedback for performance improvements in training [20, 21]. For CPR training, provider performance is bolstered when accurate and detailed information on performance quality is provided in real time. Automated feedback devices leveraging objective performance measures provide real-time audio and visual feedback about performance and lead to improved CPR quality [1, 9, 12, 14, 15, 19, 22]. This feedback increases CPR quality in both training and during actual performance of CPR [12, 23, 24]. Evidence supports the use of automated real-time visual feedback for developing skill in chest compressions [25, 26].

### Purpose

Although evidence supports brief, frequent CPR training, optimal training intervals have not been established. The purpose of the study was to compare nursing students’ CPR skills (compressions and ventilations) with 4 different spaced training intervals, with the total number of training events held constant: daily (4 days in a row), weekly (1 time per week for 4 consecutive weeks), monthly (1 time per month for 4 consecutive months), and quarterly (1 time per quarter for 4 consecutive quarters).

## Methods

### Design and sample

To examine the quality of CPR performance following variations in training intervals, we randomly assigned nursing students (n = 475) in 10 schools of nursing across the United States to 1 of 4 training intervals: daily, weekly, monthly, quarterly. To be included in the study, participants needed to be in the first year of their prelicensure nursing program and be certified in basic life support (BLS) from the American Heart Association or American Red Cross. Students were excluded from participating if they had a health condition at the time of the study that precluded their performing CPR. The study was conducted between September 2015 and June 2018. The Institutional Review Boards (IRBs) of Duke University Health System (Pro00053223), Air Force Research Laboratory (FWR20140115X), Robert Morris University (#20160106357), and Indiana University of Pennsylvania (IRB00004175) approved the study. The other schools of nursing relied on the Duke University Health System IRB approval under the IRB Authorization Agreement for an Individual Protocol. Students volunteered for the study and provided written consent to participate.

### CPR training

CPR training was performed on a Laerdal Resusci Anne^®^ QCPR^®^ adult manikin on the RQI mobile simulation station in each school’s simulation or skills laboratory. Participants used a step stool, except for a few students who were too tall to comfortably perform CPR with the stool. The training was facilitated by one or more designated nurse educators who were certified in BLS and trained in the study protocol.

In each training session, participants first had a pretest where they performed 60 compressions followed by 12 bag-mask ventilations to assess their current CPR skills. No real-time feedback was given at this time. The training sessions with the RQI are intended to be practice sessions of the discrete skills, not a sequence of CPR. The 60 compression and 12 ventilation training sessions reinforce the proper technique for performing each of these skills. Participants then watched brief demonstration videos included with the RQI program that provide an orientation to the equipment and reinforce the fundamentals of how to perform compressions and ventilations. Next, they reviewed key points on the proper technique to use for compressions and ventilations that were summarized and illustrated on laminated cards on the mobile simulation station, also provided in the RQI program. The videos were viewed on a laptop placed next to the mobile station.

Following the pretest, instructional videos, and laminated card review, participants performed 60 compressions followed by 12 bag-mask ventilations with real-time feedback turned on. They were able to view their performance on the computer screen beside the manikin, using this visual feedback to guide their depth and rate of compressions and volume and rate of ventilations. After a brief (4–10 minute) rest period, participants were tested on their skills as a posttest (same as pretest). In the pre- and posttest, students did not receive any real-time feedback on their performance from the manikin or the site educator. Each training session in each of the 4 types of training intervals (daily, weekly, monthly, quarterly) followed the same procedure.

### Measures

The outcome measures for this study were quality of compressions and ventilations as measured by the RQI program. RQI provides overall composite scores, used in this study, on the performance of compressions and ventilations using scales of 0–100%, with 75% in each skill required to pass. The overall compression score is based on mean compression depth and rate, full release of compressions, the correct number of compressions per cycle, and correct hand placement. The overall ventilation score is determined by mean ventilation volume and rate, and compliance with inspiration time [27]. The scores are calculated so that the more CPR performance deviates from AHA standards, the larger the reduction in scores. Demographic data and information about participants’ prior and current CPR training and experience were also collected.

### Procedures

**Fig 1:**
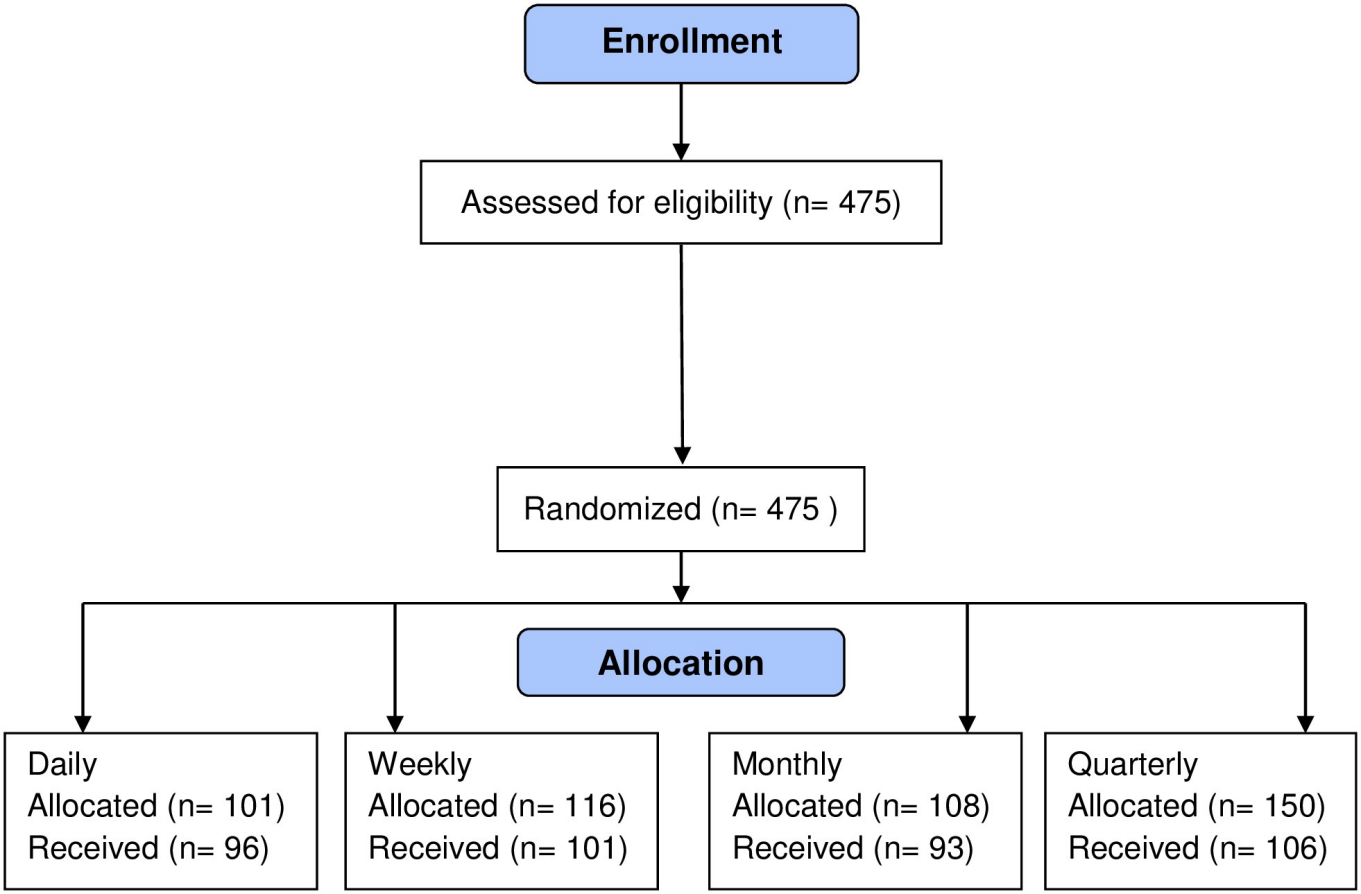
Participant flow chart.

At each school of nursing, after students agreed to participate, they were randomly assigned to a training group (daily, weekly, monthly, quarterly). The research assistant (RA) used an online random number generator to determine these groups. Our goal was to have 90 students per training group. Assuming higher attrition in the quarterly group, because it necessarily spanned multiple academic semesters and some students in this group might graduate from the nursing program before completing the study, we initially randomized 140 participants for the daily, weekly, and monthly training interval groups, and 165 for the quarterly training group. Participants were given unique ID numbers that were used to log into the RQI system and to track each participant’s performance across training sessions. One year after we started data collection, we evaluated the number of participants in each group who had completed all 4 training sessions with attrition rates per group, and adjusted the recruitment goals accordingly. After removing students who did not complete all 4 sessions, we met our goal of having 90 students/training group as listed in Fig 1.

An overall score of at least 75% in compressions was required to move on to ventilations; this same score was required for ventilations. If the minimum score was not achieved, students repeated the training with feedback a maximum of one more time. If students still did not reach the minimum score of 75% with this additional training, they remained in the study, receiving more training during their next session consistent with the RQI system, which is intended for practice of CPR to improve skills. The protocol for the study detailed steps to be followed and was provided in writing to the schools.

### Data analysis

Data on participants’ performance of CPR skills were collected through the RQI program and stored in HealthStream’s learning management system (HealthStream, Inc., Nashville, TN) and the Air Force Research Laboratory’s MindModeling system [28, 29] (https://mindmodeling.org). The RA entered the demographic data and information on participants’ experience with CPR into Research Electronic Data Capture (REDCap). All data were subsequently merged for analyses.

Data were analyzed using the R statistical software [30]. To test for differences in demographic variables among training groups, ANOVA was used for continuous measures and Chi-square for categorical measures. For each CPR performance outcome measure, differences in pretest scores were compared across sessions and training intervals (representing the daily, weekly, monthly, quarterly conditions), and were analyzed using a linear mixed model because it accounts for unequal numbers of students across conditions in a repeated measures design. Consistent with other studies in the literature [8], the analyses focused on pretest scores because we wanted to assess whether students were ready to perform compressions and ventilations following training intervals before receiving additional training. All significance testing was done at the .05 level.

## Results

There were 475 participants who enrolled in the study and were randomized into the 4 training intervals. Of that number, 396 (83.4%) completed all four training sessions: daily (n = 96), weekly (n = 101), monthly (n = 93), and quarterly (n = 106) (Fig 1). Demographic and CPR experience data were reported for 355 (there was missing data for 41 participants). The mean age of participants was 27.24 (SD 8.12) years, ranging from 18 to 61, and most were female (n = 316). Nine of the students performed CPR in an actual resuscitation attempt prior to beginning the nursing program, and during the study, 16 participants provided CPR in an actual cardiac arrest. Forty-seven students (n = 12 daily, 12 weekly, 10 monthly, 13 quarterly) had an additional CPR training experience during the study even though they were already certified in BLS. No significant differences in any of the demographic variables or in CPR experience across the training groups were found.

All of the students recruited for the study who agreed to participate, as described in the consent document, were enrolled in the study, as all met inclusion criteria: they were beginning nursing students and were BLS certified by either American Heart Association or American Red Cross. One nursing student was excluded from participating (prior to enrollment) for a health condition at the time that precluded her performing CPR.

### Effects of training intervals on quality of compressions

#### Overall compression score

**Fig 2:**
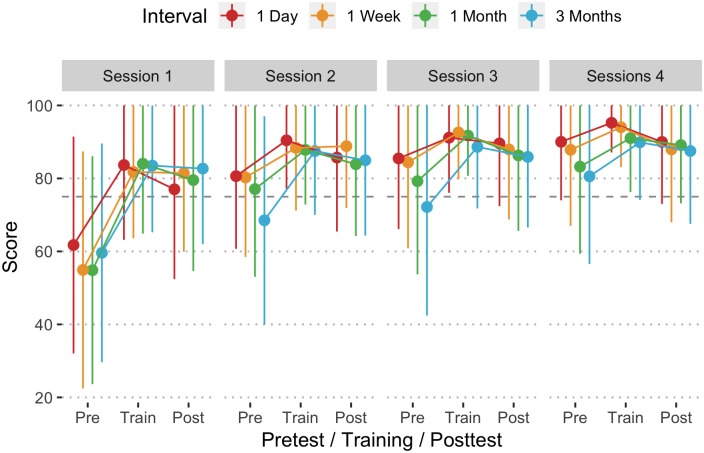
Mean overall compression score (1 SD Error Bar) by interval, pretest/training/posttest assessment, and session. Horizontal dashed line at 75 represents the minimum performance target.

Each training session consisted of a pretest to assess current CPR skills, followed by training with real-time feedback, and after a brief rest period, a posttest (same as pretest). At session 1 pretest, even though all of the participants were certified in BLS, they were not able to provide quality compressions, scoring less than the 75% criterion (Fig 2). No significant differences were observed in pretest performance across the different training intervals (F = 1.21, df = 3, 390, p >.05). For the daily training interval, pretest scores increased from a mean in session 1 of 61.7 (SD = 29.7) to a session 4 mean of 90.0 (SD = 16.0) (t = 8.52, df = 1158, p < .001). Participants in the other training intervals also had significant increases in scores across the 4 sessions (Table 1). At session 4, there was a significant difference in pretest scores between daily and quarterly training (t = 2.62, df = 1401, p = .044), but not the other conditions. Shorter training intervals resulted in larger performance increases than longer training intervals, meaning that participants acquired skill faster if time between training opportunities was shorter (F = 2.31, df = 9, 1159, p = .014).

**Table 1:** Pretest means (SD) for compression measures across sessions and intervals. Contrasts provide post hoc comparisons of session 1 and 4 for each interval, with t-values and significance levels at * *p* < .05, ** *p* < .01, and *** *p* < .001.

Interval	Session Means (SD)				Post hoc Contrast
(days)	1	2	3	4	1 x 4
*Overall Compression Score*					
1	61.7 (29.7)	80.6 (19.9)	85.5 (19.3)	90.0 (16.0)	8.52 ***
7	54.9 (32.5)	80.3 (21.8)	84.4 (23.5)	87.8 (20.8)	10.06 ***
30	54.8 (31.2)	77.1 (24.0)	79.2 (25.5)	83.2 (23.9)	8.41 ***
90	59.6 (30.0)	68.5 (28.5)	72.2 (29.8)	80.6 (24.0)	6.55 ***
*Percentage of Compressions with Adequate Depth*					
1	70.8 (29.7)	90.0 (12.4)	90.8 (16.5)	94.0 (11.7)	8.88 ***
7	66.8 (34.3)	89.3 (16.5)	94.7 (11.1)	95.3 (12.8)	11.08 ***
30	65.7 (30.9)	85.8 (19.1)	87.8 (19.8)	91.6 (15.2)	9.78 ***
90	72.4 (29.4)	83.2 (22.5)	86.5 (21.2)	90.4 (16.5)	7.22 ***
*Percentage of Compressions with Adequate Rate*					
1	44.1 (41.8)	54.7 (40.4)	70.9 (35.7)	72.7 (34.6)	5.66 ***
7	35.8 (37.6)	54.9 (43.0)	65.3 (38.0)	68.1 (36.6)	6.49 ***
30	47.2 (42.4)	48.8 (40.3)	57.0 (40.8)	65.3 (37.5)	3.53 **
90	44.6 (38.6)	44.4 (40.5)	53.5 (41.8)	61.9 (39.6)	3.57 **
*Percentage of Compressions with Adequate Release*					
1	98.9 (2.4)	98.5 (5.6)	98.5 (5.2)	98.8 (3.1)	0.17
7	98.8 (3.3)	98.7 (4.5)	98.6 (3.4)	98.1 (6.8)	1.21
30	98.5 (4.4)	98.6 (5.2)	99.0 (2.0)	99.0 (1.8)	0.97
90	98.6 (4.0)	97.5 (8.4)	98.3 (8.1)	99.0 (1.6)	0.74
*Percentage of Compressions with Correct Hand Placement*					
1	97.3 (10.0)	95.7 (15.0)	97.2 (9.7)	98.0 (8.0)	0.32
7	95.6 (13.6)	98.2 (6.0)	94.8 (17.3)	95.8 (13.1)	0.10
30	95.8 (14.8)	97.0 (12.8)	95.0 (16.4)	94.0 (18.4)	0.86
90	96.2 (13.9)	94.2 (16.8)	93.5 (18.9)	94.4 (16.5)	0.95

#### Percentage of compressions with adequate depth, rate, and release, and correct hand placement

**Fig 3:**
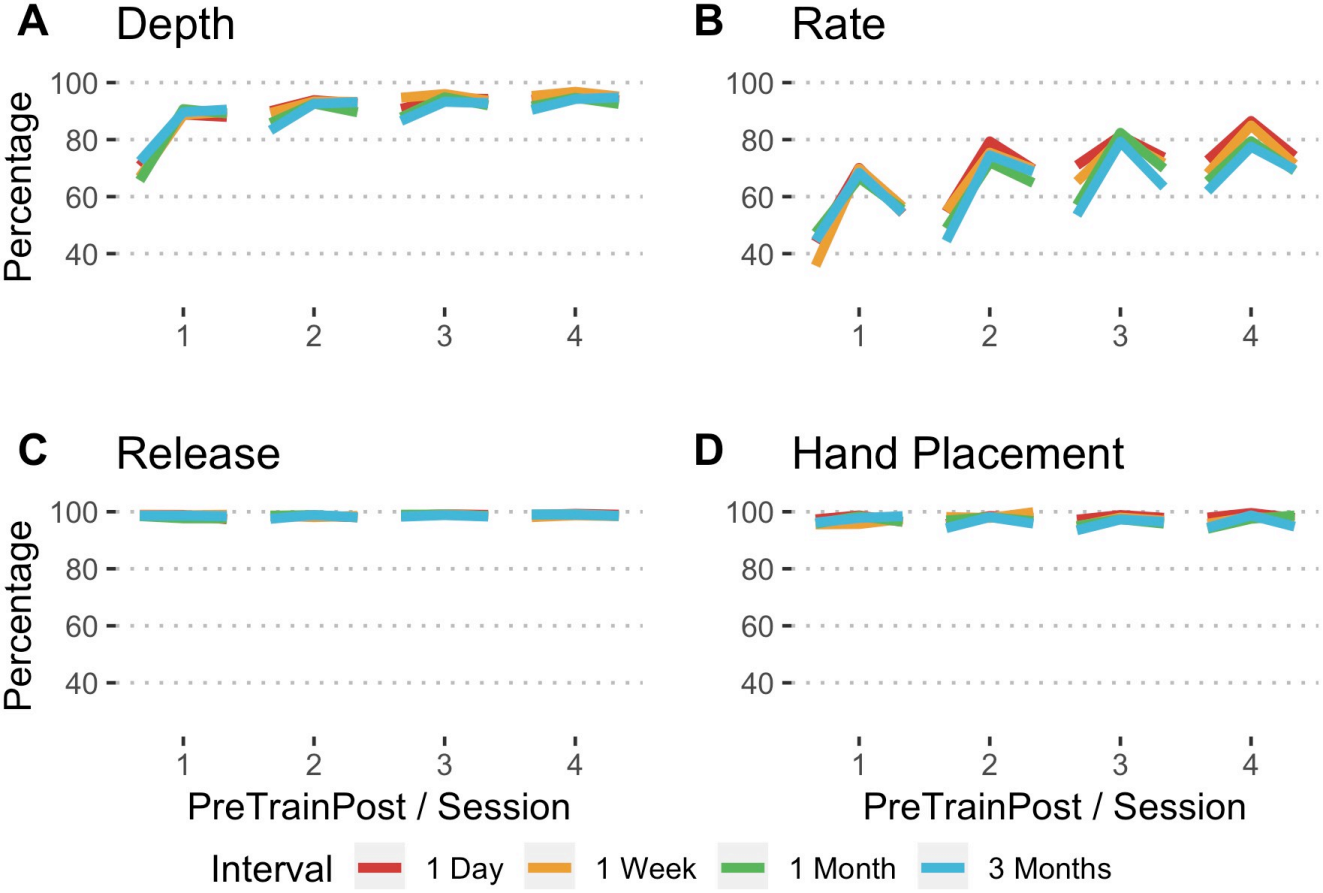
Mean percentages of compressions with adequate depth, rate, release, and hand placement by interval, session, and pretest/training/posttest assessment.

Similar to the overall compression score, pretest scores for the percentage of compressions with adequate depth increased significantly from session 1 to 4 for all intervals (Table 1, Fig 3A). However, in contrast to overall compression score, by session 4, there were no differences in students’ ability to compress with an adequate depth based on the timing of their training (F = 2.35, df = 3,378, p >.05).

Students in this study had difficulty compressing at an adequate rate. At pretest of session 1, scores for this skill ranged from 35.8 to 47.2. From session 1 to 4, the percentage of compressions with adequate rate improved significantly for all intervals (Table 1, Fig 3B). At pretest of session 4, there were no differences in scores based on training interval (F = 1.48, df = 3, 378, p >.05).

The percentage of compressions with adequate release was high for students at pretest for all intervals, and students maintained high performance across the 4 sessions (Fig 3C). The percentage of compressions with correct hand placement at pretest was consistently high as well across the 4 sessions (Fig 3D).

### Effects of training intervals on quality of ventilations

#### Overall ventilation score

**Fig 4:**
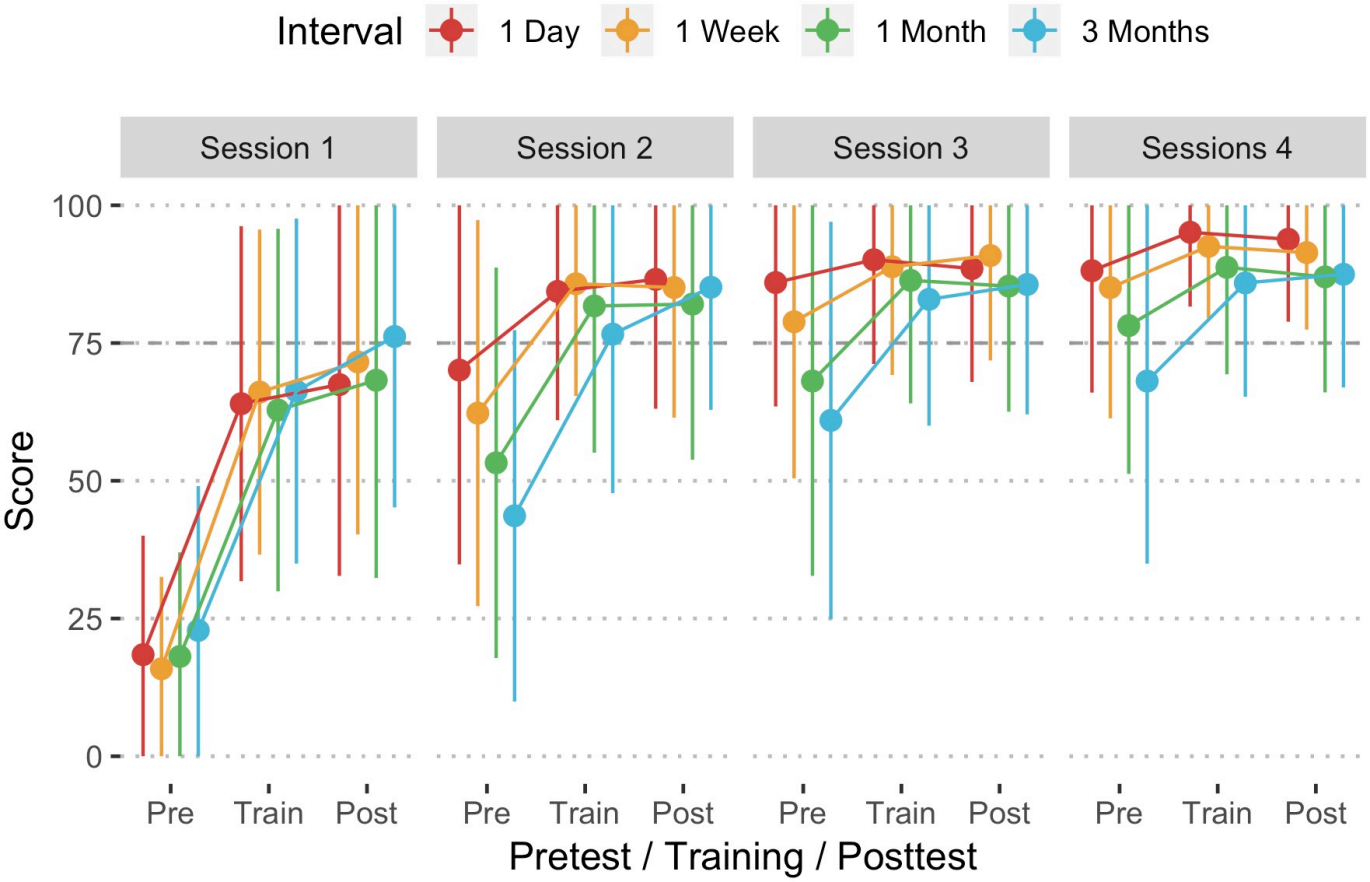
Mean overall ventilation score (1 SD Error Bar) by interval, session, and pretest/training/posttest assessment. Horizontal dashed line at 75 represents the minimum performance target.

Similar to the overall compression scores, overall ventilation scores were not significantly different at pretest of session 1 (F = 0.12, df = 3, 391, p >.05). For students who had daily training, ventilation scores increased from a pretest mean score of 18.4 (SD = 21.6) in session 1 to a mean score of 88.1 (SD = 22.1) by session 4 (t = 19.74, df = 3, 1157, p < .001). Similar increases were found for the other training intervals (Table 2, Fig 4). By the pretest of session 4, scores ranged from 68.1 (quarterly training) to 88.1 (daily training). The improvement varied by interval, with shorter intervals resulting in more improvement (F = 7.51, df = 9, 1159, p < .001). On session 4 pretests, there were significant differences in the percentage of ventilations with adequate volume between students who had training daily versus quarterly (t = 4.84, df = 237, p < .001) and weekly versus quarterly (t = 4.31, df = 1251, p < .001), but no differences among the others.

**Table 2:** Pretest means (SD) for ventilation measures across sessions and intervals. Contrasts provide post hoc comparisons of session 1 and 4 for each interval, with t-values and significance levels at * *p* < .05, ** *p* < .01, and *** *p* < .001.

Interval	Session Means (SD)				Post hoc Contrast
(days)	1	2	3	4	1 x 4
*Overall Ventilation Score*					
1	18.4 (21.6)	70.1 (35.3)	86.0 (22.5)	88.1 (22.1)	19.74 ***
7	15.9 (16.7)	62.3 (35.0)	78.9 (28.4)	85.1 (23.7)	20.17 ***
30	18.1 (18.9)	53.3 (35.4)	68.1 (35.4)	78.2 (26.9)	16.75 ***
90	22.8 (26.2)	43.6 (33.7)	61.0 (36.0)	68.1 (33.1)	13.42 ***
*Percentage of Ventilations with Adequate Volume*					
1	53.3 (37.2)	71.1 (34.0)	74.6 (33.7)	76.0 (31.4)	4.85 ***
7	55.0 (34.0)	64.4 (34.0)	72.9 (32.1)	76.7 (30.0)	4.71 ***
30	47.3 (38.1)	55.7 (35.3)	66.9 (36.3)	66.8 (35.1)	4.13 ***
90	50.6 (39.2)	49.8 (37.7)	62.5 (37.2)	61.1 (36.4)	2.40

#### Percentage of ventilations with adequate volume

**Fig 5:**
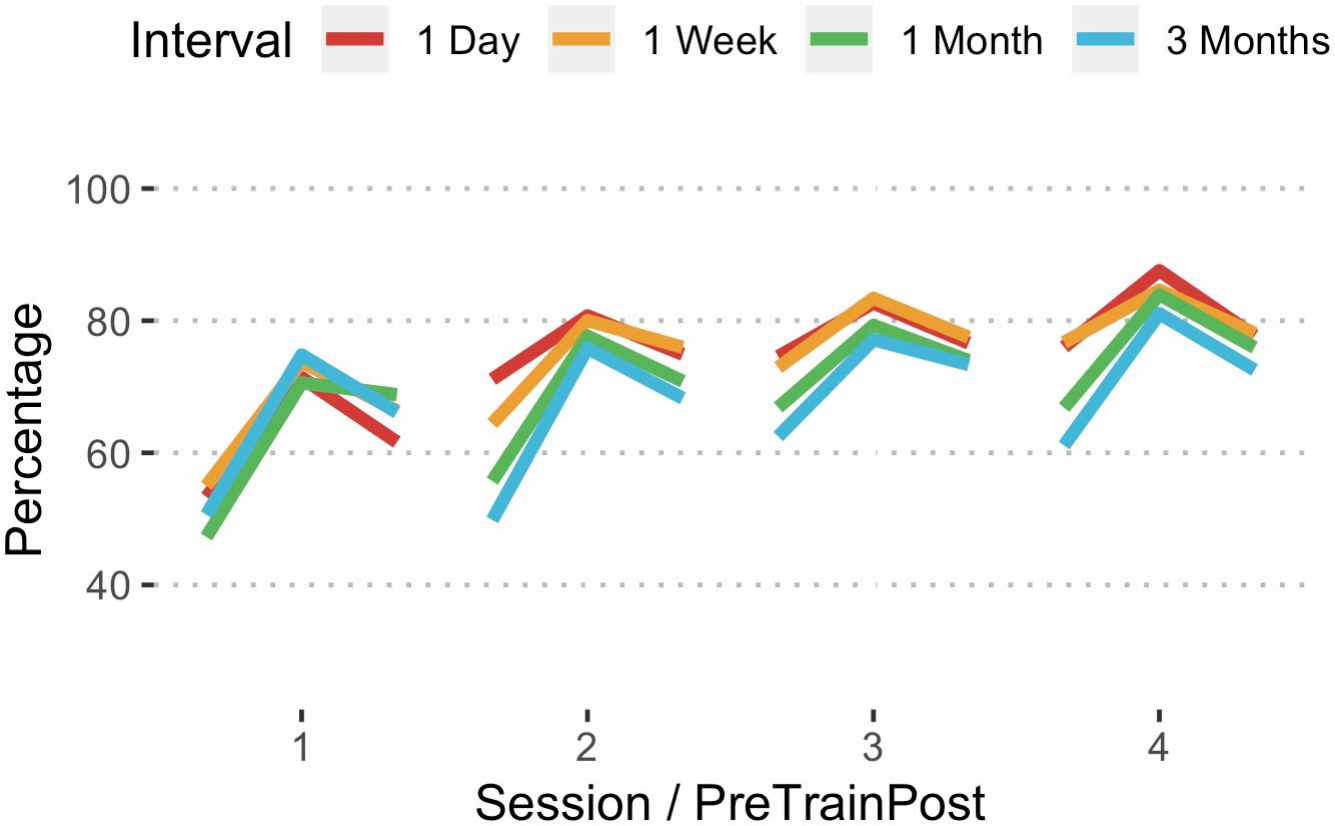
Mean percentage of ventilations with adequate volume by interval, session, and pretest/training/posttest assessment.

Scores for the percentage of ventilations with adequate volume improved from sessions 1 to 4 (Fig 5). In the daily, weekly, and monthly intervals, this improvement was statistically significant, but not for the quarterly (Table 2). By session 4 there were significant differences between the daily and quarterly (t = 2.90, df = 1440, p = .020), weekly and quarterly (t = 3.06, df = 1447, p = .012), but not among the others.

## Discussion

Although all of the students who participated in this study were certified in BLS, few were able to adequately perform compressions and ventilations at pretest, consistent with earlier studies [1, 8, 11–15]. Overall compression scores improved from sessions 1 to 4 in all training intervals (all p < .001), but shorter intervals (daily training) resulted in larger increases in compression scores by session 4. There were similar findings for ventilation skills, but at session 4, both daily and weekly intervals led to better skill performance.

With a one-time training in BLS, learners may know “what to do” at the time of the training, but need an opportunity for continued practice to gain proficiency and be able to perform skills at a later time [1, 12, 31, 32]. As participants practiced CPR in each subsequent training session, and received real-time feedback on performance, they developed better skills than in the prior session. These findings are consistent with other studies involving varied health care providers. In a study in a community hospital in the United States, practice of CPR skills using the RQI program improved compression and ventilation skills of nurses, physicians, and other providers [33]. Participants who trained daily had the highest scores in all 4 sessions, followed by students in the weekly training group, and students who trained quarterly had the lowest scores. By session 4, there were significant differences between daily and quarterly training with the shortest training interval resulting in the largest increase in overall compression and ventilation scores.

Other studies have demonstrated the value of shorter spacing on CPR training. For example, Anderson and colleagues [8] found that nurses who trained monthly had better CPR performance than those who trained every 3, 6, and 12 months. Multiple studies support frequent training intervals for CPR skill development with varied health care providers [1, 12, 15, 17–19]. Our study, however, appears to be the only one that has examined the effects of daily and weekly CPR training. For students and others learning to perform CPR, the opportunity to train on consecutive days or weeks may be beneficial: if learners are aware of specific errors in performance, it may be easier for them correct performance and refine skills where there is less time in between practice sessions. A policy implication may be that if the sole interest is in minimizing the time required to achieve proficiency, then training intervals as short as daily may be advisable. An opportunity to master CPR skills quickly, with shorter time between training sessions, followed by distributed practice might be the most effective for developing and maintaining CPR skills over time.

### Limitations

There were missing demographic and CPR experience data for 41 participants. In addition, 47 of the students, spread similarly across the training intervals, had an additional CPR training session during the study, which we did not consider in the analysis. Participants in our study were nursing students at the beginning of their program, potentially affecting generalizability of the findings to other health care providers. However, in many clinical settings, providers are trained in BLS on an annual basis and rarely, if ever, use those skills or practice them beyond this annual training. As a result, their skill level may be similar to students. Although daily or even weekly training intervals might be best for mastering CPR skills as a beginning learner, retention was not the focus of this study. Retention of skills at one year should be examined in a future study. In addition, there are cost and practical considerations in arranging training of providers on a daily basis.

## Conclusions

CPR performance improved in all 4 training intervals, though shorter intervals resulted in a faster overall rate of improvement for overall compression and ventilation skills. With shorter intervals between refresher training opportunities, there is less opportunity for knowledge and skill decay. If students and others learning to perform CPR are aware of specific errors in performance, provided via real-time feedback during training, it may be easier for them to correct performance and refine skills when there is less time in between practice sessions.
